# Predicting HLA CD4 Immunogenicity in Human Populations

**DOI:** 10.3389/fimmu.2018.01369

**Published:** 2018-06-14

**Authors:** Sandeep Kumar Dhanda, Edita Karosiene, Lindy Edwards, Alba Grifoni, Sinu Paul, Massimo Andreatta, Daniela Weiskopf, John Sidney, Morten Nielsen, Bjoern Peters, Alessandro Sette

**Affiliations:** ^1^Division of Vaccine Discovery, La Jolla Institute for Allergy and Immunology, La Jolla, CA, United States; ^2^Instituto de Investigaciones Biotecnológicas, Universidad Nacional de San Martín, Buenos Aires, Argentina; ^3^Department of Bio and Health Informatics, Technical University of Denmark, Kongens Lyngby, Denmark; ^4^University of California San Diego, La Jolla, CA, United States

**Keywords:** HLA, immunogenicity, immunodominance, epitopes, predictions, bioinformatics, TCR repertoire

## Abstract

**Background:**

Prediction of T cell immunogenicity is a topic of considerable interest, both in terms of basic understanding of the mechanisms of T cells responses and in terms of practical applications. HLA binding affinity is often used to predict T cell epitopes, since HLA binding affinity is a key requisite for human T cell immunogenicity. However, immunogenicity at the population it is complicated by the high level of variability of HLA molecules, potential other factors beyond HLA as well as the frequent lack of HLA typing data. To overcome those issues, we explored an alternative approach to identify the common characteristics able to distinguish immunogenic peptides from non-recognized peptides.

**Methods:**

Sets of dominant epitopes derived from peer-reviewed published papers were used in conjunction with negative peptides from the same experiments/donors to train neural networks and generate an “immunogenicity score.” We also compared the performance of the immunogenicity score with previously described method for immunogenicity prediction based on HLA class II binding at the population level.

**Results:**

The immunogenicity score was validated on a series of independent datasets derived from the published literature, representing 57 independent studies where immunogenicity in human populations was assessed by testing overlapping peptides spanning different antigens. Overall, these testing datasets corresponded to over 2,000 peptides and tested in over 1,600 different human donors. The 7-allele method prediction and the immunogenicity score were associated with similar performance [average area under the ROC curve (AUC) values of 0.703 and 0.702, respectively] while the combined methods reached an average AUC of 0.725. This increase in average AUC value is significant compared with the immunogenicity score (*p* = 0.0135) and a strong trend toward significance is observed when compared to the 7-allele method (*p* = 0.0938). The new immunogenicity score method is now freely available using CD4 T cell immunogenicity prediction tool on the Immune Epitope Database website (http://tools.iedb.org/CD4episcore).

**Conclusion:**

The new immunogenicity score predicts CD4 T cell immunogenicity at the population level starting from protein sequences and with no need for HLA typing. Its efficacy has been validated in the context of different antigen sources, ethnicities, and disparate techniques for epitope identification.

## Introduction

The identification of T cell epitopes has an important implication in several immunological contexts spanning from vaccine design to diagnostics in cancer, allergies, and infectious diseases fields. Most of the epitope identification is currently performed using bioinformatics prediction systems aimed to identify T cell immunogenicity and also to dissect the mechanisms underlying development of T cell responses. Currently, the majority of the T cell prediction methods are based on prediction of HLA binding affinity, which is a key requisite for human T cell immunogenicity. However, there is a lack of effective strategies able to predict immunogenitcity at the population level, which is of particular importance when HLA typing data are not available. To overcome this issue, it is important to identify the common HLA binding affinity characteristics able to distinguish immunogenic peptides from non-recognized peptides. Two main classes of HLA molecules are important in the immunological context. Class I molecules presents epitopes to CD8 T cells, while class II molecules present epitopes to CD4 T cells. Prediction of HLA class I binding has reached high accuracy with area under the ROC curve (AUC) values greater than 0.9 (1–7), similarly HLA class II predictions have significantly improved in the most recent years reaching significant levels of accuracy (with AUC values in the range of 0.760–0.870) (8–10). However, HLA molecules are highly polymorphic and epitope prediction at the population level has to take into account this high level of heterogeneity.

We previously shown that in the case of HLA class I, focusing on 25–30 main HLA A and B allelic variants provides coverage of a large fraction of the general population (11). Similarly, in the case of HLA class II, about 40–50 allelic variants provide coverage of most frequent allelic variants (12). Prediction of HLA binding is usually performed with allele-specific algorithms, since binding motifs of different HLAs are rather diverse. However, in the case of HLA class II, it is also noted that a high degree of overlap exists between the epitope binding of different variants (13). Indeed, it was shown that the epitopes dominantly recognized are often capable of binding to many different HLA class II alleles. These epitopes (named promiscuous epitopes) account for 50% or more of the total responses at the population level (14).

The “7-allele method” was specifically optimized for prediction of HLA class II responses at the population level (15) based on the prediction of promiscuous epitopes. While this method is associated with significant predictive value, it is also expected that many of the peptides that are predicted or experimentally shown to bind HLA class II molecules may not induce T cell responses. This is because although HLA binding is necessary it is not sufficient by itself for T cell immunogenicity. Other factors such as antigen processing and the size of the TCR repertoire capable of recognizing any given MHC/epitope complex are key factors in ultimately determining immunogenicity (16–18).

In particular, it has been shown that the TCR repertoire is a key factor in shaping epitope immunodominance (19–23). In the case of HLA class I, different algorithms have been devised that evaluate a peptide sequence for the presence of certain amino acids, presumably interacting with TCRs, as a contributing factor to epitope’s intrinsic immunogenic potential (24–26).

In the present study, we evaluate an approach to predict HLA class II immunogenicity at the population level, regardless of specific HLA haplotype, by training neural networks (NNs) with well-characterized sets of immunogenic epitopes dominant in general human populations. This approach could thus probe not only the influence of HLA binding but also potentially detect factors beyond HLA class II binding that would be encoded in the primary sequence of potential epitopes.

## Materials and Methods

### Datasets

The datasets used for training were derived entirely from experimental data generated in our laboratory using congruent techniques as a mean to rely on tightly controlled datasets. In addition, we also utilized epitopes that were associated with positive tetramer data as part of the training, because tetramer data are regarded as “gold standard” of quality and specificity in analyzing T cell response. Conversely, the datasets used for validation were derived from scientific literature using a broad variety of techniques and antigens, and generated from different laboratories worldwide. This choice was made to ensure the robustness of the validation provided.

#### Training Dataset Assembly

We used 15-mer peptides derived from several datasets described in peer-reviewed articles or obtained by in-house studies following same experimental approach (Table 1). In some cases, the epitope sets were selected based on interim analysis and do not exactly match the final epitope lists in the published articles. The peptides were tested for immune recognition in cohorts of 5–150 donors by ELISPOT assays for one of the following cytokines: IFNγ, IL-5, IL-17, or IL-10. A full list of these epitopes is described in Table S1 in Supplementary Material. In total, 1,032 epitopes were selected as positives in this study. Negative peptides were selected from the same datasets listed in Table 1 following specific criteria: peptides should be negative in all tests, only peptides from proteins with at least one positive peptide recognized were included. In addition, any peptide tested more than once (due to several studies testing antigens/allergens from the same organism) giving opposite responses for the same donor was removed from the dataset. Overall, 5,739 negative peptides (Table S2 in Supplementary Material) were obtained. In some cases, set-specific adjustments in the criteria were necessary for technical reasons, as detailed below.

**Table 1:** Full list of datasets used in this study.

**(A) Training datasets**					

**Antigen (s)**	**Peptide selection method**	**# of donors**	**Reference**	**# of epitopes**	**# of control peptides**

*Mycobacterium tuberculosis*	Overlapping	18	(27)	65	53
	Predicted	28	(28)		1,043
	Overlapping	61	(29)		362
	Confirmed epitopes	61	(29)		137
Timothy grass	Overlapping	25	(14)	60	360
	Predicted	35	(30)		360
	Overlapping	21	(31)		6
	Overlapping	37	(32)		0
House dust mite (HDM)	Overlapping	20	(32)	52	6
Cockroach	Overlapping	19	(33)	71	521
Dengue antigens	Predicted	150	(34)	325	140
Erythropoietin	Overlapping	5	(35)	9	11
CRJ1 and CRJ2	Overlapping	54	(36)	30	18
Mouse allergens	Predicted	22	(37)	82	885
Novel HDM antigens	Predicted	20	(38)	105	186
Pertussis vaccine antigens	Overlapping	53	(39)	100	202
Ragweed allergens	Overlapping	25	(40)	15	183
Tetanus		20	(41)	28	98
ZIKA virus polyprotein	Overlapping	18	(Grifoni et al., unpublished)	48	529
Yellow fever virus polyprotein	Overlapping	42	(Weiskopf et al., unpublished)	42	639
Overall				1,032	5,739

**(B) Validation dataset derived from literature**					

**Antigen (s) (***species***)**	**# of donors**	**Reference**	**# of epitopes**	**# of control peptides**	

Acetylcholine receptor subunit alpha (*Homo sapiens*)	22	(42)	4	18	
Circumsporozoite (CS) protein (*Plasmodinium vivax and falciparum)*	22	(43)	4	4	
Conserved hypothetical lipoprotein (*Francisella tularensis*)	10	(44)	3	10	
Other protein (*Plasmodium falciparum*)	12	(45)	7	5	
CS protein (*Plasmodium falciparum*)	64	(46)	7	10	
CS protein (*Plasmodium falciparum*)	35	(47)	7	7	
Api m 1 (*Apis mellifera*)	40	(48)	6	9	
Myelin basic protein (*Homo sapiens*)	12	(49)	3	3	
CS protein (*Plasmodinium vivax*)	52	(50)	7	5	
Acetylcholine receptor sub. γ and δ (*Homo sapiens*)	22	(51)	14	42	
Acetylcholine receptor sub. α (*Homo sapiens*)	22	(52)	8	17	
Glutamate decarboxylase 2 (*Homo sapiens*)	44	(53)	2	10	
Structural polyprotein (*Rubella virus*)	10	(54)	4	7	
Envelope glycoprotein D (*Human herpesvirus 1*)	24	(55)	6	6	
Thyroglobulin and thyrotropin receptor (*Homo sapiens*)	15	(56)	5	10	
Fusion glycoprotein F0 (*Morbillivirus*)	13	(57)	12	50	
Poa p 5, *Poa pratensis* (*Kentucky bluegrass*)	13	(58)	9	8	
Myelin basic protein (*Homo sapiens*)	20	(59)	6	7	
Structural polyprotein (*Rubella virus*)	14	(60)	4	74	
Acetylcholine receptor sub. δ and α (*Homo sapiens*)	58	(61)	12	33	
Hev b 1 (*Hevea brasiliensis*)	19	(62)	2	2	
Api m 1 (*Apis mellifera*)	10	(63)	7	6	
TRAP (*Plasmodinium falciparum*)	50	(64)	21	30	
Nucleoprotein (*Morbillivirus*)	19	(65)	9	40	
Genome polyprotein (*Hepatitis C virus*)	22	(66)	14	13	
Subtilisin-like protease 6 (*Trichophyton rubrum*)	38	(67)	8	20	
Blood groups Rh(D) and Rh(CE) polypeptides (*Homo sapiens*)	22	(68)	19	15	
Myelin proteolipid and myelin basic protein (*Homo sapiens*)	16	(69)	7	14	
Polyprotein Ent. virus B; Glut. Decarboxylase2 (*Homo sapiens*)	22	(70)	7	26	
Gal d 1 (*Gallus gallus*)	14	(71)	2	1	
Genome polyprotein (*Hepatitis C virus*)	10	(72)	5	122	
Hev b 6 (*Hevea brasiliensis*)	16	(73)	4	12	
Bos d 9 (*Bos Taurus*)	10	(74)	2	5	
Cha o 1 (*Chamaecyparis obtusa*)	19	(75)	10	24	
Genome polyprotein (*Hepatitis C virus*)	22	(76)	12	257	
Genome polyprotein (*Hepatitis C virus*)	41	(77)	18	33	
Bos d 9, *Bos taurus* (*Bos Taurus*)	29	(78)	8	12	
Cytochrome P450 2D6 (*Homo sapiens*)	80	(79)	28	29	
Capsid protein VP1 (*Human parvovirus*)	19	(80)	8	54	
Integrin beta-3 (*Homo sapiens*)	31	(81)	7	51	
Genome polyprotein (*Hepatitis C virus*)	44	(82)	7	286	
Equ c 1 (*Equus caballus*)	10	(83)	15	32	
Merozoite surface protein 1 (*Plasmodium falciparum*)	48	(84)	10	18	
Cry j 1 (*Cryptomeria japonica*)	12	(85)	4	33	
Cha o 2 (*Chamaecyparis obtusa*)	19	(86)	6	36	
Capsid protein VP1 (*Adeno-associated virus*)	16	(87)	28	62	
Non-specific lipid-transfer protein (*Prunus persica*)	15	(88)	3	5	
Aquaporin-4 (*Homo sapiens*)	32	(89)	6	10	
UniProt:B8ZU53 (*Mycobacterium leprae*)	152	(90)	8	1	
Pas n 1 allergen (*Paspalum notatum*)	18	(91)	4	11	
Pen a 1 allergen (*Farfantepenaeus aztecus*)	16	(92)	15	13	
Genome polyprotein (*Tick-borne encephalitis virus*)	47	(93)	26	46	
Other wolf or dog protein (*Canis lupus*)	25	(94)	18	12	
Can f 5 (*Canis lupus*)	24	(95)	25	31	
Botulinum neurotoxin type A (*Clostridium botulinum*)	25	(96)	6	13	
Genome polyprotein (*Rhinovirus A and C*)	20	(97)	15	34	
Botulinum neurotoxin type A (*Clostridium botulinum*)	14	(98)	6	14	
Overall			530	1,758	

##### *Mycobacterium Tuberculosis* (TB) Antigens

We selected 65 previously known 15-mer epitopes identified from the vaccine candidate antigens and that captured 80% of the response (27–29).

##### Timothy Grass (TG) Known Allergens

Previous studies identified 20 epitopes that accounted for 79.5% of the total response to a set of TG-derived pollen antigens (Phl p allergens) in TG allergic individuals (14, 31, 32). Most of the datasets are composed by 15-mers as they were based on HLA class II binding prediction (15, 99). However, since some of those epitopes were not 15-mers, to compare those with the rest of the dataset longer epitopes were dissected into the composing 15-mers and each 15-mers belonging to the longer peptides has been classified as a positive, with the same process being used for negative peptides. In addition, 19 peptides were described to cover an NTGAp19 peptide pool, which were selected to encompass at least 40% of the total IL-5 response directed against all NTGA peptides screened (30).

##### House Dust Mite (HDM) Allergens

The peptide set included the 34 most dominant epitopes cumulatively accounting for 90% of the total allergen-specific response detected in our screen (32). Analogous to the TG set, longer regions were deconstructed into 15-mers, which yielded 52 peptides in total.

##### Cockroach (CR) Allergens

71 most dominant epitopes were selected based on total spot forming cells (SFC) values greater than 1,000 (33).

##### Dengue (DENV) Antigens

Peptides predicted to bind various frequent DRB1 alleles were tested in about 10 HLA-matched donors. The sets comprised 325 epitopes, positive in at least two donors with PBMC derived from normal blood donors from the Colombo (Sri Lanka) region that were seropositive for DENV antibodies and thus representative of natural infection (34). Negative peptides were those tested in at least 10 donors and found to be uniformly negative.

##### Erythropoietin

Tangri et al. screened overlapping peptides and reported nine epitopes recognized by at least 40% donors (35).

##### CRJ1 and CRJ2 Japanese Cedar Allergens

This set contained overlapping 15-mers spanning the CRJ1 and CRJ2 allergens (36). We selected 30 dominant epitopes based on average response magnitude of >100 SFC (sum of IL-5 and IFNγ) in either of two group of allergic donors: those who lived in Japan for extended periods of time and USA sensitized donors who had not lived in Japan. A total of 18 control negative peptides were derived from allergens CRJ1 and CRJ2 and selected based on a response frequency of one donor or less and an individual SFC response <100 SFC.

##### Mouse Allergens

Peptides derived from mouse allergens, largely selected by the 7-allele algorithm were tested in 22 donors (37). A total of 89 dominant epitopes were defined on the basis of total SFC >150 and recognized in at least two donors.

##### Novel House Dust Mite Antigens

The peptides screened were predicted with the 7-allele method from 96 HDM (novel and known) proteins in 20 HDM allergic donors (38). We selected the 106 more dominant epitopes, recognized in multiple donors and with an overall magnitude of >300 SFC total (accounting for about 50% of the total response).

##### Pertussis Vaccine Antigens

The peptide set was comprised of 16-mers overlapping by eight residues, spanning the entire sequence of the antigens. We selected the top 100 epitopes recognized in at least 4 of the 53 total donors analyzed, and accounting for approximately 75% of the total response (39).

##### Ragweed Allergens

This set included 16-mers overlapping by eight amino acids, spanning the entire sequence of the antigens (40). A total of 15 epitopes accounting for 75% of the total response was selected. If variants were present, the most common variant was selected.

##### Tetanus Toxoid (TT) Antigen

We selected a set of 28 epitopes, recognized in at least 2 out of 20 donors tested (41), and predicted by the 7-alleles method (15). As a control, we selected a set of 57 peptides, which were studied but not recognized, neither in the Immune Epitope Database (IEDB, www.iedb.org) nor in the study by Antunes et al. (41), and an additional set of 41 peptides that were not predicted by the method and also neither recognized in the study by Antunes et al. nor identified in the IEDB as positive human responses. In the case of the third set of 41 peptides, there were 261 15-mers in the Tetanus set. Among them 124 were predicted to be binders with predicted 7-allele median percentile rank ≤20.0. Out of the 137 non-predicted peptides, those with predicted 7-allele median percentile rank >40.0 were selected (67 peptides) for screening to be included in non-predicted AND non-epitope set. From this list, we eliminated peptides that were overlapping by more than five AA residues with any of the epitope (recognized in our study or annotated as positive in IEDB). The remaining 41 peptides were included in the set of “control peptides” that were not predicted and neither recognized in the Antunes et al. study nor identified as positive response in IEDB.

##### ZIKA Virus (ZIKV) Antigens

A set of 15-mer peptides spanning the entire sequence of the ZIKV proteome was tested with a 14 days re-stimulation protocol in 18 donors. A total of 48 epitopes were defined as being positives in at least two donors (Grifoni et al., unpublished).

##### Yellow Fever (YF) Antigens

The set of epitopes tested includes 94 previously described YF CD4 T cell epitopes with known HLA class II restriction (IEDB) and sets of peptides predicted to bind different HLA DRB1 molecules. CD4+ T cells from 42 donors vaccinated with YF17 vaccine were co-cultured with autologous antigen-presenting cells and HLA-matched YF DRB1 predicted peptides. After 14 days, IFNγ response against individual peptides was determined as previously described (100). Epitopes were defined as peptides eliciting an SFC of 664 SFC/10^6^ or more. This resulted in the identification of 42 unique peptides (Weiskopf et al., unpublished).

#### IEDB Validation Datasets

To generate additional datasets to evaluate the performance of the various predictive schema, we sought to identify literature records reporting overlapping peptide studies. Accordingly, we queried the IEDB for papers which contained both positive and negatives records curated in the paper, related to HLA class II restricted T cells. This query identified 870 papers; which were further refined by filtering by “overlapping” mentioned in the abstract, resulting in 183 records.

The abstracts of those records were manually inspected, to select papers truly related to study of immunogenicity of overlapping peptide sets. At this stage, we excluded records relating to Phl p, TT, TB (already represented in the previous sets) and studies based on transgenic mice to obtain 102 relevant papers.

We next removed papers where the peptide size was less than 15, or where less than 10 donors were studied (resulting in 82 papers). Each of these 82 papers was manually inspected and additional papers were discarded upon manual inspection for a variety of reasons, including the paper not reporting testing for full sets of overlapping peptides, ambiguous reporting of negative results or peptide size tested, no clear discrimination between positive and negative responses, testing pools of peptides with no deconvolution, and similar problems.

This resulted in a final selection of 57 papers (Table 1). For each paper, based on the data disclosed and on the author’s interpretations, we captured the most dominant epitopes accounting for the majority of responses and/or consistently positive in multiple donors. We selected peptides that were consistently negatives as corresponding negative controls. In studies where large numbers of donors were tested and essentially all peptides were positives, we selected the peptides positives in one or more donors. A list of PUBMED Ids, and the criteria used to select the “top” epitopes and the “bottom” negative controls is provided in Table S3A in Supplementary Material. A list of positive and negative control peptides is provided in Table S3B in Supplementary Material.

#### Tetramer Training Dataset

A dataset corresponding to epitopes described as positive in tetramer staining experiments was downloaded from IEDB (accessed June 2015) (101) using the following selection criteria: “*Positive Assays Only, Epitope Structure: Linear Sequence, T Cell Assays: qualitative binding/multimer/tetramer (tetramer), No B cell assays, No MHC ligand assays, MHC Restriction Type: Class II, Host Organism: Homo sapiens (human) (ID:9606, human)*.” The exported dataset was filtered keeping only 15-mer epitopes for which a source antigen protein ID was available. For each unique positive peptide, we took its source protein sequence using the antigen genome ID and scanned that protein for all possible 15-mers overlapping by 10 amino acids. The original positive peptide was then considered as an immunogenic one and the rest of the obtained peptides were used as negatives. The tetramer dataset had 124 unique positives and 5,319 negatives that are presented in Table S4 in Supplementary Material.

### Artificial Neural Network (ANN)-Based Predictions Using *NNAlign* Method

The NN training for peptide sequences was performed using the *NNAlign* method (102). The method uses classified peptide data for training and identifies nested shorter sequence patterns that constitute an informative motif to separate positive from negative examples. As an input to *NNAlign*, we used sequences of our 15-mer peptides and their assigned observed immunogenicity score (1.0 for immunogenic and 0.0 for non-immunogenic). The method was trained using extensive cross-validation where part of the data is left out of the training process and is used for evaluation purpose only. For each peptide, the method returns a predicted score between 0.0 and 1.0, with high values identifying more immunogenic peptides and low values non-immunogenic peptides. The *NNAlign-1.4* software package was downloaded from http://www.cbs.dtu.dk/services/NNAlign/. The method was trained for each possible motif length varying from 1 to 15. The data for cross-validation was split based on common motifs within peptides with a maximum overlap to nine and varying the motif length. Input peptides were encoded using Sparse and BLOSUM schemes. No rescaling was done to the input data. We also chose to preserve repeated flanks in the original data and do not realign networks with offset. The method was trained with 5 hidden neurons using 10 seeds for each network architecture. It is possible that other encoding approaches, choice of NN design, or choice of other learning algorithms could have let to better results, but such a comparison was outside the scope of our current manuscript.

### Receiver Operating Characteristic (ROC) Curves and AUC Values

To measure how different approaches are capable of classifying peptides into epitopes and non-epitopes, ROC curves were used (103). Varying cutoff for predicted scores, peptides were classified into immunogenic and non-immunogenic and the numbers of true positives (TPs) and false positives (FPs) were obtained. The ROC curve was made by plotting TP rate as a function of FP rate at each cutoff. AUC is a useful measure for assessing predictive performance of a prediction method. AUC values range from 0.5 to 1, where 0.5 corresponds to random and 1 to perfect predictions. The AUC value can be interpreted as the probability that the predicted score for a randomly chosen immunogenic peptide is higher than the score of a randomly chosen non-immunogenic peptide.

### HLA Binding Predictions

We utilized the previously described 7-allele method (15) to derive HLA binding propensities. The 7-allele method predicts immunogenicity based on the median percentile predicted binding of seven alleles representative of the binding motifs most commonly recognized in the general human population, and is available on the IEDB website (104).

### Generation of Two-Sample Logo

The two-sample logo was created with 15-mer peptides (15 residues from N-terminal were extracted in case of longer peptides) from all the datasets combined, for epitopes and non-epitopes. For two-sample logo, both epitopes and non-epitopes datasets (in FASTA formatted files) were submitted to the online tool (http://www.twosamplelogo.org/cgi-bin/tsl/tsl.cgi) with default settings except for *p*-value, which was set to 0.01 and resolution of 600 dpi (105).

### Statistical Analysis

The statistical analysis was performed using Prism 7 (Graph-Pad Software, San Diego, CA, USA). The non-parametric Wilcoxson matched-pair signed rank test with method of Pratt was utilized to assess the significance differences between sets of different AUC values.

## Results

### Derivation and Validation of an ANNs-Derived Immunogenicity Score

We assembled T cell epitope datasets from different previously published peptide screening studies performed in our laboratory (Table 1). In all cases, peptides were screened using ELISPOT assays to detect which peptides stimulated secretion of cytokines. Table 1 summarizes the number of donors that were screened for each peptide set and if the peptides were selected to overlap specific antigens, or if they were selected based on predicted binding affinity. Dominant epitopes accounting for a majority of the T cell responses as described in more detail in the Section “Materials and Methods” were considered positives (Table S1 in Supplementary Material, *N* = 1,032 peptides). Peptides that did not give any response in any donor but that came from proteins for which at least one peptide was positive, were considered negatives (Table S2 in Supplementary Material, *N* = 5,739 peptides). This additional criterion for negative classification was used to ensure that the lack of recognition was not simply due to lack of availability of the source protein necessary for antigen presentation.

**Figure 1:**
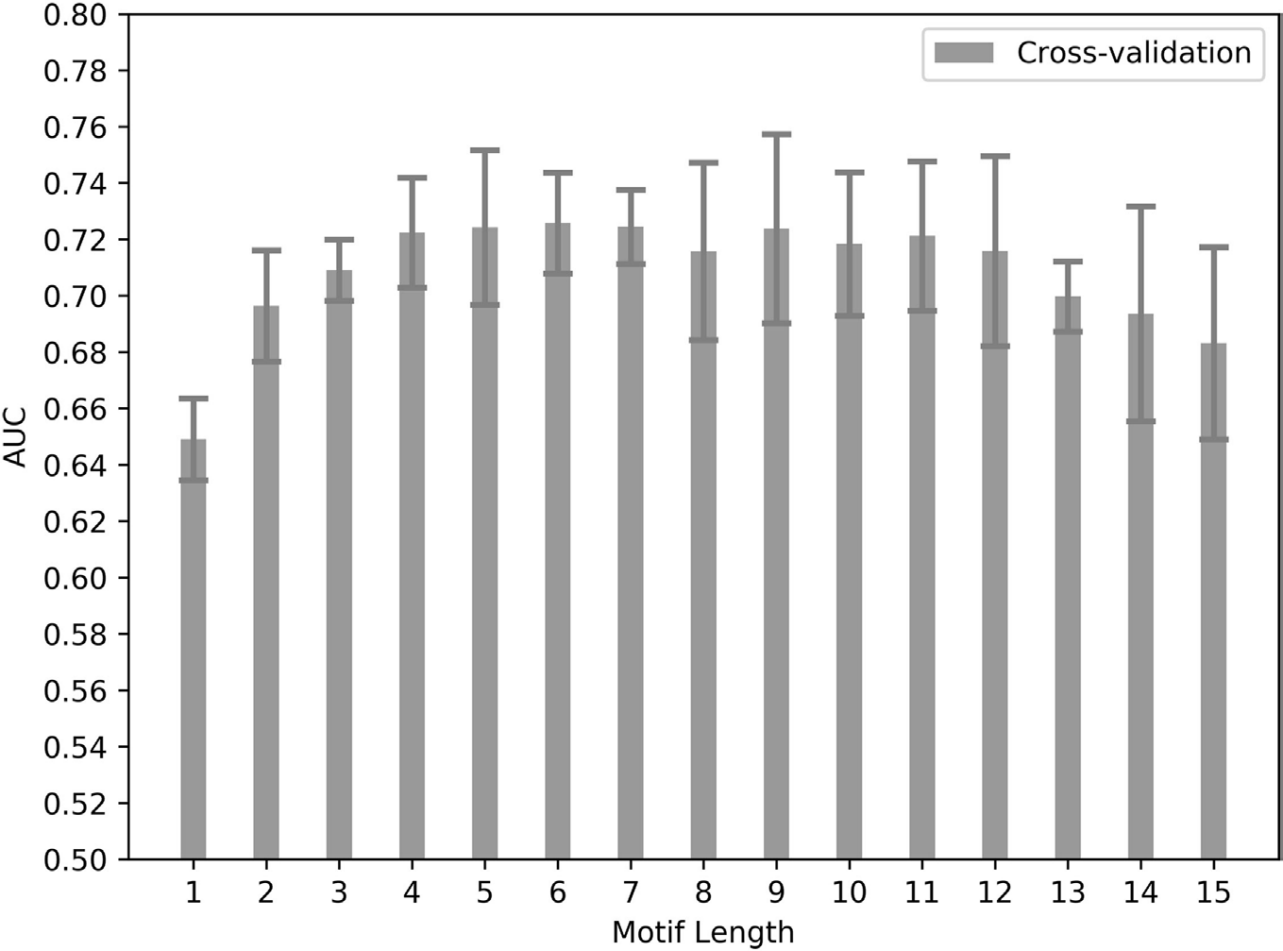
Predictive performances for different motif lengths. Bars show cross-validation performance for the training dataset. Area under the ROC curve (AUC) values are shown for each artificial neural network training done by choosing different sequence lengths to define a preferred sequence motif within a 15-mer peptide. Error bars show SD of the five cross-validation sets.

This initial dataset was used to train an ANN-based method called *NNAlign* (102). The *NNAlign* method takes an unaligned peptide set and aims to find a linear sequence core within the peptides, which differentiates the positive (immunogenic) from the negative (non-immunogenic) peptides. The length of the sequence core was varied systematically from a single residue to 15 residues immunogenicity score (ranging 0–1) for each variation is retrieved, and prediction quality was assessed using fivefold cross-validation. Several sequence core lengths showed AUC values greater than 0.7 which is generally considered as a good prediction quality value, suggesting that the ANNs shows differences between positive and negative peptides based on the peptide motif. In terms of sequence motif length, the cross-validation did not indicate a clear optimal length, as the prediction performance was similar for motif lengths between 3 and 12 (Figure 1). A motif length of nine residues is consistent with the known size of peptide core region engaging HLA and TCR. For this reason, a motif length of nine was selected for the following analyses.

### Combining Immunogenicity and HLA Binding Predictions

To consider both HLA binding and the immunogenicity prediction (which presumably incorporate the capacity of being recognized by TCR), we combined our ANN-based immunogenicity predictions with HLA class II binding predictions. Only one method has been described to predict epitopes based on HLA binding at the population level, namely the 7-allelle method, which was previously empirically optimized based on immunogenicity datasets (15).

To combine immunogenicity and HLA binding scores, we used the median percentile rank score (*HLA_score*) of the 7-allele method (ranging from 0 to 100) and combined it with our NN-based immunogenicity score after converting it to a percentile score, so that it would also range from 0 to 100 and could be comparable with the HLA_score, using the formula (*Imm_score*) = (1 − neural network immunogenicity) × 100. The two scores were combined as follows:

(1) Combined score=α× Imm score+(1−α)×HLA score.

Next, we systematically varied the value of α in the interval of 0 ≤ α ≤ 1. From the equation above, when α = 1 the results depend only on the immunogenicity predictions by the NN, while with α = 0 only HLA binding predictions are used to define immunogenicity.

**Figure 2:**
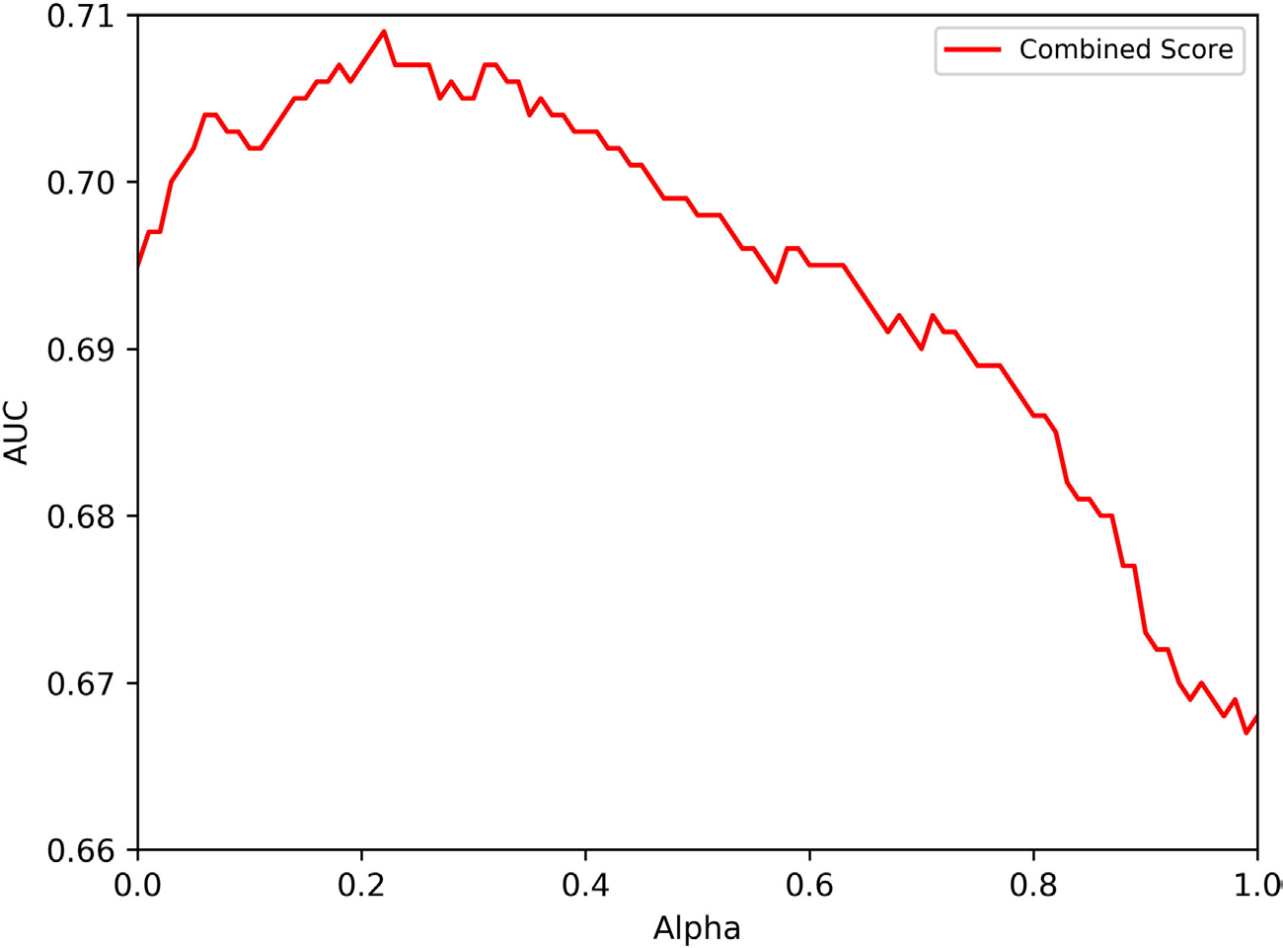
Predictive performances obtained combining HLA binding and immunogenicity scores. The figure shows the performance dependency on an α coefficient used to combine HLA binding and immunogenicity scores. The model trained on the training dataset described in the text and validated on independent literature datasets, also described in the text.

To assess the performance of the immunogenicity score, the 7-allele method and their combination, we used independent literature-based datasets. Specifically, we searched the IEDB for papers which described results of testing overlapping peptide sets related to human HLA class II restricted T cells. These epitope sets thus represent a broad range of studies, representing a “real-life” portrait of epitope identification studies performed in the worldwide scientific community. These epitope sets are listed in Table S3A in Supplementary Material and described in more detail in the Section “Materials and Methods” and the sequences are provided in Table S3B in Supplementary Material. Overall, a total of 57 different sets derived from independent literature studies were curated, entailing a total of 530 positive and 1,758 negative peptides. Figure 2 depicts the predictive performance of the combined score, displaying the average of the different AUC values obtained for each of the different datasets. The 7-allele method was associated with AUC values of 0.695, and the immunogenicity score was associated with an average AUC value of 0.670. In terms of combination of the two algorithms, the performance increased and reached a peak at 0.71 for an α value of 0.50.

### Performance of the Immunogenicity Score, Eliminating Redundancy Between Training and Testing Datasets

It is expected that inclusion of additional data points would increase the performance of an NN model. Accordingly, we incorporated an additional dataset of CD4 T cell epitopes identified by tetramer mapping studies. We reasoned that this would provide high quality epitopes since the tetramer-staining assay is commonly considered a “gold standard” assay for epitope characterization. The dataset was obtained by querying the IEDB for 15-mer peptides that were tested positive by tetramer staining assays. For each positive peptide, its source protein was scanned for 15-mer peptides overlapping by 10 amino acids, with the positive peptide sequences being removed and the remaining peptides used to construct a negative dataset. The final tetramer dataset is composed of 124 unique positives and 5,319 unique negative peptides (Table S4 in Supplementary Material).

The datasets utilized to train and evaluate the NN models contained some redundancies, which could affect the evaluation and inflate performance. To avoid this issue, we eliminated any redundancy between the training set (Table 1 and tetramer set combined) and the validation set of the 57 independent studies (Table 1) by filtering out any peptide sharing a common 9-mer sequence.

**Figure 3:**
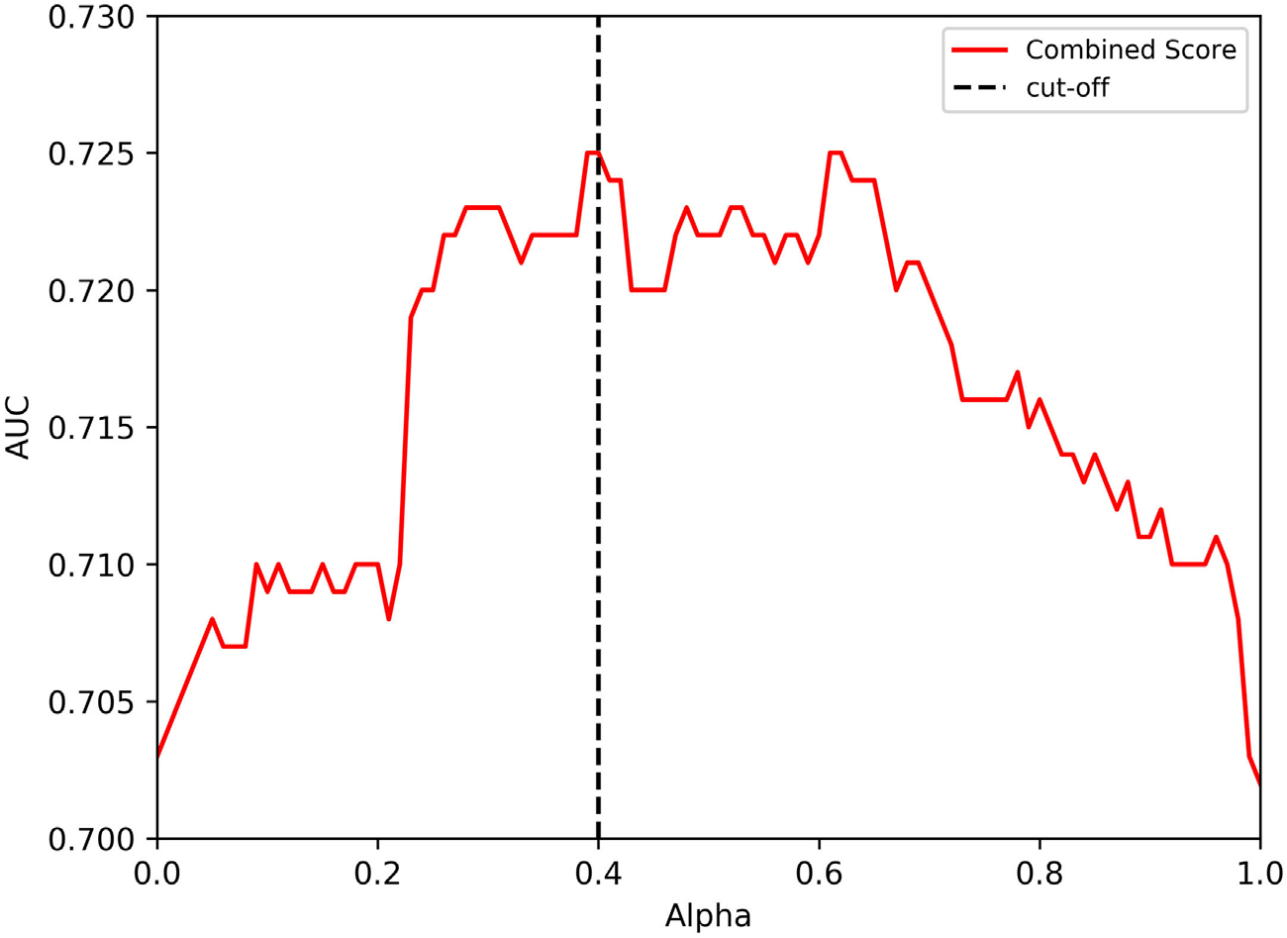
Performance of independent literature datasets with combined approach and varying degree of alpha on the model trained with initial, in-house and tetramer datasets. The prediction values from HLA score and immunogencity score using different values of alpha are shown. A cutoff of 0.4 value for alpha is also highlighted by a dotted line.

In the analysis performed, a clear optimal alpha was not observed. The data in Figure 2 seemed to indicate an optimal alpha around 0.2–0.3, while the analysis from Figure 3 indicates two optimal peaks at about 0.4 and 0.6. Since the data in Figure 3 are inherently more reliable because of training with a higher number of data points, we empirically selected 0.4 as the alpha to include in the next set of analyses. When this analysis was performed, the 7-allele method prediction and the immunogenicity score were associated with similar performance (average AUC values of 0.703 and 0.702, respectively) while the combined methods again afforded gain in performance, reaching an average AUC of 0.725 (Figure 3). This increase in average AUC values of the combined methods is significant when compared with the average AUC values of the immunogenicity method with a *p* value of 0.0135 using Wilcoxon matched-pairs signed rank test, and a strong trend toward significance when compared to the 7-allele method with a *p* value of 0.0938. These results, together with the ones obtained with the tetramer dataset confirm that both the 7-allele and the immunogenicity score method had significant predictive value on their own which are in both cases enhanced by their combination.

### Two-Sample Logo of a General Immunogenicity Motif

**Figure 4:**
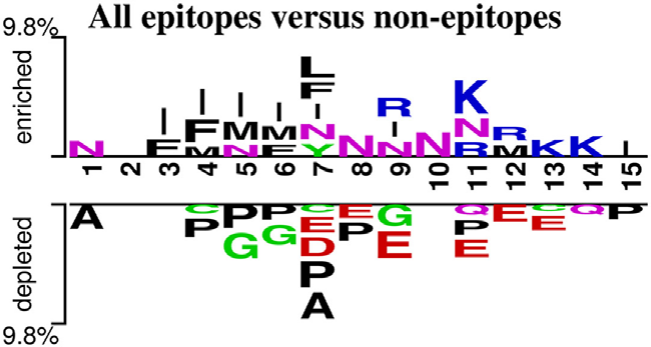
Two-sample logo created using epitopes and non-epitopes in all the data (*p*-value < 0.01). The immunogenicity motifs for epitopes and non-epitopes were derived from the combination of all the datasets.

Next, we analyzed the epitopes and non-epitopes from all the datasets combined for their positional residue conservation and plotted two-sample logo using 15 residues from the N-terminus of the peptides (105) (Figure 4). The two-sample logo represents amino acids which were significantly different in epitopes and non-epitopes based on *p*-value (<0.01) calculated using *t*-test. Amino acid residues enriched in the epitope dataset are mostly positively charged, while amino acid residues depleted in the epitope dataset (and enriched in the non-epitope dataset) are mostly negatively charged. In other words, epitopes have higher numbers of positive charged residues like arginine (R) or lysine (K) at positions 9th and 11th–14th, whereas non-epitopes contained aspartate (D) and glutamate (E) at positions 7th–9th and 11th–13th. A preference for hydrophobic residues is also observed [such as proline (P) and alanine (A)] in non-epitopes, whereas isoleucine (I), phenylalanine (F), and asparagine (N) are enriched in the epitope set. To further address the significance of the logo, we split the dataset into five sets, where each set contains 80% of the total dataset, the results in Figure S1 in Supplementary Material confirm that the most prevalent feature revealed by the logo are in consistent with the two-sample logo created using whole dataset (Figure 4). These results suggest that some of these preferences may be contributing to T cell recognition or MHC binding or represent a result of processing enzymes. These possibilities will be addressed in future studies.

### Epitope Prediction Threshold and Implementation of an Online Tool

**Figure 5:**
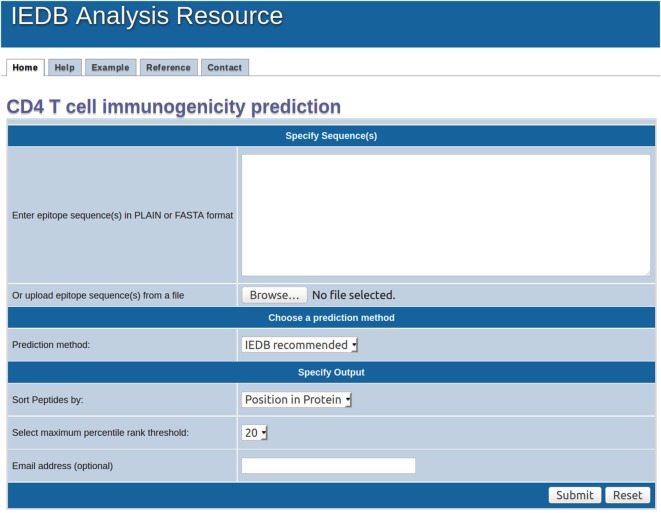
Screenshot for home page of immunogenicity prediction server.

We next determined the performance of the combined score using different cutoff values ranging from 0 to 100 (Table 2) for each study. To this end, we calculated the performance of overlapping datasets derived from literature at different threshold settings using the percentile combined score at α = 0.4. As a first step, for each study we calculated the numbers of: true negative (TN) defined as non-immunogenic peptides predicted as non-immunogenic, FP defined as non-immunogenic peptides predicted as immunogenic, false negative (FN) defined as immunogenic peptides predicted as non-immunogenic, and TP defined as the immunogenic peptides predicted as immunogenic. Based on these values we calculated sensitivity [= (TP/TP + FN) × 100] and specificity [= (TN/TN + FP) × 100]. Finally, we determined that cutoff values of 8, 36, and 66 allowed capturing, respectively, 20, 51, and 75% of the epitopes with a corresponding specificity of 91, 65, and 37%. We also estimated the fraction of peptides needed to test in order to observe a defined fraction of epitopes using the following formula: [(TP + FP)/(TP + TN + FP + FN)] × 100. A value of 43 was associated with equal sensitivity and specificity (59). To make this approach user friendly, we also implemented an online version of this algorithm (Figure 5). The tool is freely available in the IEDB website at http://tools.iedb.org/CD4episcore/.

**Table 2:** Performance of overlapping dataset derived from literature at different threshold settings using the percentile combined score.

Threshold	Average sensitivity	Average specificity	Total peptide to be synthesized (average)
8	20	91	13
18	31	85	21
36	51	65	39
43	59	59	46
66	75	37	68

*The performance for each study is calculated individually and then averaged*.

*Sensitivity = (TP/TP + FN) × 100, specificity = (TN/TN + FP) × 100, % total peptide to be synthesized = [(TP + FP)/(TP + TN + FP + FN)] × 100*.

*TN, true negative; FP, false positive; FN, false negative; TP, true positive*.

## Discussion

Bioinformatics predictions to identify T cell epitopes are frequently used in the context of designing and testing vaccines and diagnostics for infectious diseases, allergies, and cancer. While several HLA allele-specific predictive algorithms (10) and T cell epitopes predictive strategies based on MHC class II binding have been described (106–108), development of effective strategies to predict immunogenicity at the population level are lacking and remain therefore of significant interest. This is important, since in the real-life applications most often encountered HLA typing data is often unavailable.

Here, we report an approach to identify sequence motifs distinguishing immunogenic peptides recognized by CD4+ T cells from non-recognized peptides, independent of the restricting HLA class II allele. We confirm that the previously described 7-allele method (15) is effective in predicting epitopes and could narrow the range of peptides to be used for biological testing. Importantly, we find significant improvements of a combined HLA binding + immunogenicity approach over immunogenicity predictions alone and a strong trend toward significance of a combined HLA binding + immunogenicity approach over HLA binding predictions alone.

The machine learning algorithm we applied (*NNalign*) was developed to identify sequence motifs of a specific length that distinguish peptide sets—in our case immunogenic from non-immunogenic peptides. We found that motif lengths between 8 and 11 residues gave the best performance in the classification of the different datasets. This motif length is in line with what has been described with epitope residues in contact with the T cell receptor, and the length of the epitope core binding characteristic of the HLA class II molecule, which is also about nine amino acids long (20, 109).

The fact that the increase over predictions performed on HLA binding alone is rather small suggests, in line with previous studies, that HLA binding is a dominant force in shaping the repertoire of T cell epitopes. It is also possible, however, that this relatively small increase might be related to coordinate evolution between HLA binding and antigen processing and TCR recognition as suggested before by other studies (110).

Since the method was derived on immunogenicity outcomes only, it is possible that the motif defined herein is not only related to HLA binding but also incorporates overall preferences for TCR residue contacts. However, given the unbiased nature in which it was derived, it cannot be ruled out that the method may also reflect completely different processes, such as modulation by HLA-DM or increase in HLA binding stability over affinity is the actual source of the motif (111).

The predictive ability of very short motifs (3, 4 residues) is striking. Potential structural or mechanistic bases for this could be reflective of dominant influence of short stretches of residues incorporating dominant residues for HLA binding in close proximity to residues also dominant in TCR recognition (15). Examining the residues in the motif suggests that peptides with small amino acid side chains are avoided in the middle of the motif, while residues with longer side chains are overrepresented. This is qualitatively similar to what we had previously found for HLA class I restricted epitopes, and which has been reported in experimental studies using single residue substitutions (112, 113). This further supports that the motif identified coincides with properties of peptides more likely to engage a TCR. The F, M, L enrichment in the positions close to the N-terminus maybe at least in part corresponding to the P1 anchor of the MHC-II, which has similar specificity in several loci and allelic variants.

Our results have been trained over an extended set of data, derived from different methodologies and from populations of diverse ethnicities, and related to infectious diseases, allergy, and autoimmunity. The tetramer-trained algorithm seems to perform better, despite a bias toward certain HLA alleles and possible inclusion of many epitopes in negative set (i.e., other epitopes from the same protein other than the tetramer considered). We speculate that this may be due to the fact that tetramer epitopes represent usually dominant epitopes which in turn have been shown to correspond to promiscuous HLA binders. Overall, the combined training sets corresponded to over 14 thousand peptides, from over 300 different antigens and tested in over 2,500 different human donors. We believe this is an important aspect of our study, as it ensures that our building model (as related to both the 7-allele method, the immunogenicity score and the combined approach) are valid irrespective of antigen source, different ethnicities and disparate techniques for epitope identification. Our prediction method may be useful for generating off the shelf vaccine peptide libraries for pathogens or common tumor markers. Conversely, this method may be useful for an optimum selection of peptides covering individualized tumor derived neo-epitopes after NGS sequencing in HLA-typed individuals.

The algorithm is available on the IEDB website (101), and we estimate that the use of the combined immunogenicity score and 7-allele method will allow capturing 50% of the total epitopes by synthesis of 24% of the total possible overlapping 15-mers. This would translate in coverage of a 300 residues protein with 72 15-mer peptides. Future improvements of T cell epitope predictions may benefit from the increased availability of large scale datasets of peptides eluted from HLA class II molecules, datasets of specific TCRs recognizing epitopes, and datasets unraveling the role of mediators in the MHC class II processing pathway such as HLA-DM.

Even with this approach the AUC values are lower than for MHC-I analysis (1). However, it should be kept in mind that these AUC values refers to prediction at the population level encompassing T cell with diverse restriction, while the higher AUC values for MHC-I usually refers to allele-specific predictions. However, the application of the current approach from MHC-II to MHC-I, faces specific challenges. In MHC-I it is thought there is much more HLA-specific selection of epitopes, arguing against a straightforward application of the current approach, but it is possible that the alpha analysis could identify any HLA-independent components. Finally, it will be of interest to develop a similar approach to develop HLA agnostic predictors of HLA class I epitopes. Recent data suggest that it is possible to empirically develop HLA class I epitope “megapools” that afford coverage of general populations, irrespective of ethnicity (114, 115). Future studies will be focused on similar methods for HLA-agnostic prediction of class I restricted epitopes.

## Ethics Statement

Human data have been previously published and extracted from IEDB database (www.IEDB.org).

## Author Contributions

SD, EK, LE, SP, MA, and JS compiled and analyzed the data. SD, EK, AS, and BP wrote and edited the manuscript. AG and DW contributed the data. AS, MN, and BP conceived and supervised the project.

## Conflict of Interest Statement

The authors declare that the research was conducted in the absence of any commercial or financial relationships that could be construed as a potential conflict of interest. The handling Editor declared a past co-authorship with the authors.

## Supplementary Material

Table S1 List of epitopes used as a positive dataset for the training set.

Table S2 List of control peptides used as negative data for the training set.

Table S3 Validation dataset description. (a) List of papers and corresponding number of peptide as positive, negative, and intermediate immunogenicity. (b) List of positive and negative peptides for the corresponding papers.

Table S4 Additional training dataset from tetramer staining assays.
