# Supplementary material for: Predicting HLA CD4 Immunogenicity in Human Populations
Source: Front Immunol. 2018 Jun 14;9:1369. doi: 10.3389/fimmu.2018.01369 (PMC6010533; doi:10.3389/fimmu.2018.01369)
Supplement: Supplementary file 5 [file image_1.PDF]

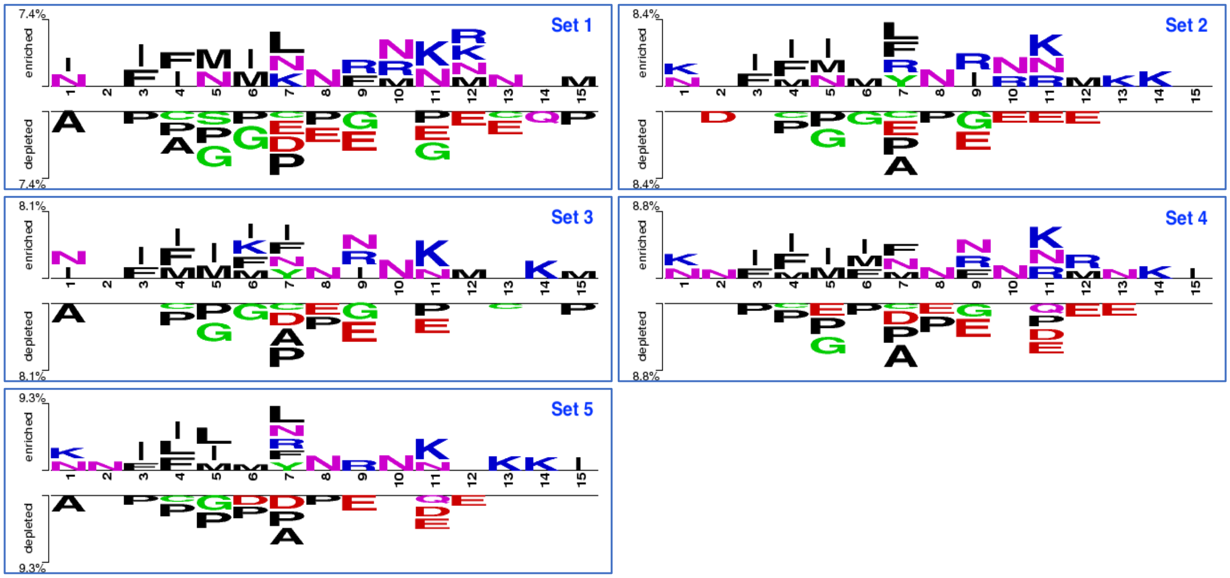

Figure S1: Two-sample logos created after splitting the complete dataset in to five sets (set1 – set 5). Each set contains 80% of the data.
